# Run-Off Replication of Host-Adaptability Genes Is Associated with Gene Transfer Agents in the Genome of Mouse-Infecting *Bartonella grahamii*

**DOI:** 10.1371/journal.pgen.1000546

**Published:** 2009-07-03

**Authors:** Eva C. Berglund, A. Carolin Frank, Alexandra Calteau, Olga Vinnere Pettersson, Fredrik Granberg, Ann-Sofie Eriksson, Kristina Näslund, Martin Holmberg, Hillevi Lindroos, Siv G. E. Andersson

**Affiliations:** 1 Department of Molecular Evolution, Evolutionary Biology Centre, Uppsala University, Uppsala, Sweden; 2 Department of Medical Sciences, Section for Infectious Diseases, Uppsala University Hospital, Uppsala, Sweden; Université Paris Descartes, INSERM U571, France

## Abstract

The genus *Bartonella* comprises facultative intracellular bacteria adapted to mammals, including previously recognized and emerging human pathogens. We report the 2,341,328 bp genome sequence of *Bartonella grahamii*, one of the most prevalent *Bartonella* species in wild rodents. Comparative genomics revealed that rodent-associated *Bartonella* species have higher copy numbers of genes for putative host-adaptability factors than the related human-specific pathogens. Many of these gene clusters are located in a highly dynamic region of 461 kb. Using hybridization to a microarray designed for the *B. grahamii* genome, we observed a massive, putatively phage-derived run-off replication of this region. We also identified a novel gene transfer agent, which packages the bacterial genome, with an over-representation of the amplified DNA, in 14 kb pieces. This is the first observation associating the products of run-off replication with a gene transfer agent. Because of the high concentration of gene clusters for host-adaptation proteins in the amplified region, and since the genes encoding the gene transfer agent and the phage origin are well conserved in *Bartonella*, we hypothesize that these systems are driven by selection. We propose that the coupling of run-off replication with gene transfer agents promotes diversification and rapid spread of host-adaptability factors, facilitating host shifts in *Bartonella.*

## Author Summary

Emerging infectious diseases represent an increasing human health problem with many examples of disease outbreaks caused by transmissions from animals to humans, such as, most recently, the bird flu virus. Genes involved in virulence and antibiotic resistance are often carried by mobile elements like plasmids and viruses, which mediate transfer between cells at an amazing speed. Rodents represent a major carrier of infectious agents, and it is therefore particularly important to study the gene transfer processes in bacteria that use rodents as their natural host reservoir. We have studied the genome of a bacterium that is naturally adapted to mice and identified many more putative host-interaction genes than were observed in previously recognized human pathogens. Furthermore, most of these genes are located in a segment of about 25% of the genome, which was massively amplified and packaged into viral particles. This is the first demonstration of targeted packaging of a portion of the bacterial chromosome into viral particles, and we propose that this is a novel strategy for increased exchange of genes involved in the infectious process.

## Introduction

Horizontal gene transfer contributes to phenomena such as pathogen emergence and antibiotics resistance, with major implications for human health. However, little is known about the mechanisms and ecological factors that influence the intensity of horizontal gene transfer in bacteria adapted to wild host populations. With about 170 cases of emerging infectious diseases during the last two decades, more than half of which are caused by bacterial infections [1], a better understanding of the mechanisms of transfer in natural isolates and how the spread of the mobile gene pool depends on environmental variables such as population size, number of infected hosts and transmission dynamics is needed.

*Bartonella* is a particularly good model system for studies of the mechanisms whereby genes are transferred in wild host populations and the selective forces leading to fixation of the transferred genes in the population. The genus consists of circa 20 described vector-borne species that infect erythrocytes and endothelial cells of mammals [2], including rodents that are major carriers of infectious disease agents. Four genomes have been sequenced to date, from *Bartonella quintana*, the agent of trench fever [3], *Bartonella bacilliformis,* the agent of Carrion's disease (unpublished), the cat-adapted *Bartonella henselae* which causes cat-scratch disease during incidental infection in human [3] and the rat-associated *Bartonella tribocorum* [4]. With single, circular chromosomes in the 1.4–2.6 Mb range, *Bartonella* species have the most highly reduced genomes in the bacterial order Rhizobiales of the alpha-proteobacteria.

A recent genomic survey identified many putative host-adaptability genes essential for bloodstream infection of *B. tribocorum* in rats [4]. The experimentally best characterized such genes in *B. quintana, B. henselae* and *B. tribocorum* are those coding for the type IV (VirB, Trw) and type V (adhesins, autotransporters) secretion systems. The VirB system translocates effector proteins into the cytoplasm of endothelial cells, thereby mediating anti-apoptotic activity and angiogenic reprogramming [5]–[7], whereas the Trw system is required for invasion of erythrocytes [8] and displays diversifying selection on the pilus proteins [9]. The *Bartonella* adhesin (BadA) is involved in binding to endothelial cells, autoaggregation, and mediates a proangiogenic response in *B. henselae* [10],[11]. The homologous proteins in *B. quintana* have essentially the same function, and are required for bloodstream infection, but have been termed Vomps (variably expressed outer membrane proteins) since they have been shown to undergo sequence and expression variation in a monkey model system [12]–[14]. Less is known about the autotransporters, however they are known to be upregulated during infection of endothelial cells [15] and may be involved in adhesion to host cells [16].

Several of the genes for secretion systems are located in a dynamic region of the genome, up to several hundred kb in size, which is thought to have originated by the integration of an auxiliary replicon [3]. DNA from this region was highly amplified in *B. henselae* upon prolonged growth [17]. Microarray hybridizations suggested bi-directional replication starting from a region encoding a few phage genes [17]. A similar phenomenon has also been observed in lambdoid phages of *Salmonella* [18], and has been termed escape replication or run-off replication. It has been suggested to arise from defects in prophage excision [18],[19]. Despite the replication of large amounts of the bacterial chromosome, the prophage that induced escape replication in *Salmonella* produced phage particles that only contained phage DNA [18].

Bacteriophage particles have been identified in several species of *Bartonella* [20]–[24] and growth experiments have suggested phage induction and lysis of *B. henselae* cells as they enter stationary phase [23]. While the morphology of the bacteriophage particles differed slightly – phages from *B. bacilliformis* and *Bartonella vinsonii* ssp. *berkhoffi* were tailed, whereas those from *B. henselae* and *B. quintana* lacked tails – they all contained 14 kb linear, double-stranded DNA, packaged in a round to icosahedral head of 40–80 nm in size [21],[22],[24]. No one has been able to fully characterize the 14 kb band, however it has been suggested to consist of random or heterogeneous bacterial DNA [21],[22],[24].

To learn more about the mechanisms and selective forces driving run-off replication, we determined the genome sequence of *Bartonella grahamii* strain as4aup, and studied the DNA content of bacteriophage particles from this species. *B. grahamii* has been isolated from several species of mice and voles and is likely to be one of the most prevalent *Bartonella* species in wild rodents [25]–[28]. It is transmitted by the rodent flea [29] and has been involved in two reported cases of human disease [30],[31]. The sequenced strain was isolated from a wood mouse (*Apodemus sylvaticus*) in central Sweden [25]. Through its broad host range, *B. grahamii* has access to a large gene pool. Since our sequenced isolate has undergone minimal cultivation in the laboratory, we expect it to represent a *Bartonella* genome in nature, with intact accessory DNA.

We demonstrate that the highly dynamic region of the chromosome, that contains many gene clusters for secretion systems, is extensively amplified and packaged into bacteriophage particles. This is the first report that associates run-off replication with bacteriophage particles. We propose that the combination of these two systems promotes diversification and rapid spread of selectively favored host-adaptability genes within and among *Bartonella* populations, facilitating host shifts.

## Results

### Comparative Genomics of *Bartonella*

#### Genome organization

**Figure 1:**
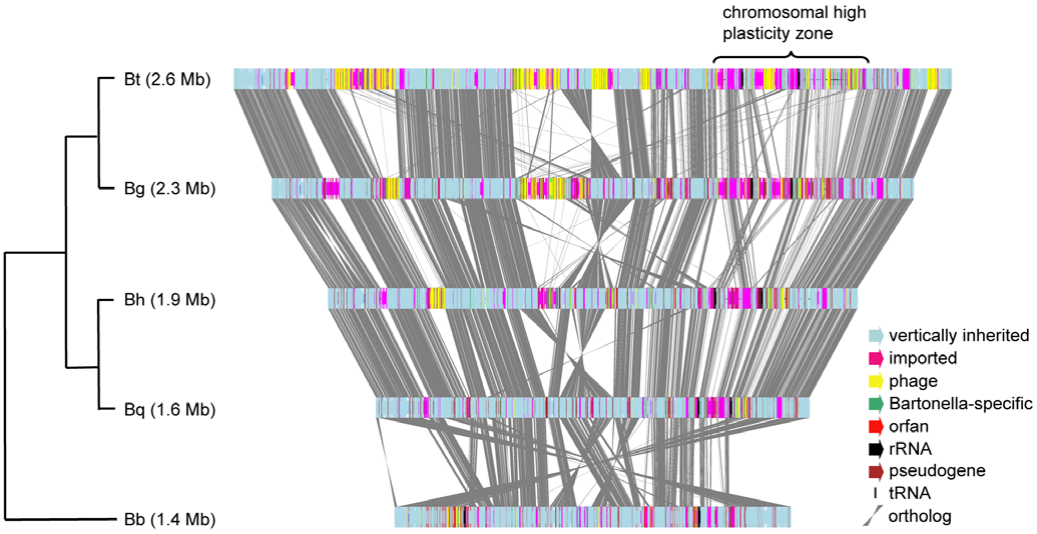
Comparison of the structures of the *Bartonella* genomes. A schematic illustration of the phylogenetic relationship of the five sequenced *Bartonella* species is shown to the left of a linear representation of their genomes. The total size of each genome is shown within parenthesis. Genes are color-coded based on phylogenetic classifications and annotation. Grey lines between genes indicate orthology. Bt: *B. tribocorum,* Bg: *B. grahamii*, Bh: *B. henselae,* Bq: *B. quintana,* Bb: *B. bacilliformis.*

*B. grahamii* strain as4aup contains a single circular chromosome of 2,341,328 bp with a predicted set of 1737 genes (Table 1). Additionally, 31 genes were identified on a plasmid of 28,192 bp, called pBGR3 in analogy with two plasmids detected in another strain of *B. grahamii* [32]. We annotated 236 sequences as pseudogenes, most of which are remnants of full-length *B. grahamii* genes. The five sequenced *Bartonella* genomes are largely collinear albeit with inversions across the terminus of replication in all lineages (Figure 1). A segment downstream of and including the second rRNA operon is inverted across *ori* in *B. bacilliformis*.

**Table 1:** Genome characteristics of *B. grahamii*.

Genome size chromosome (bp)	2341328
pBGR3 size (bp)	28192
Protein-coding genes (chromosome)	1737
Protein-coding genes (pBGR3)	31
Pseudogenes (chromosome)	236
tRNA	42
rRNA	6
tmRNA	1
Orthologs *Bartonella* a	1245
Orthologs *B. tribocorum* b	1237
Orthologs *B. henselae* b	1135
Orthologs *B. quintana* b	1042
Orthologs *B. bacilliformis* b	955

a The number of *B. grahamii* genes with an ortholog in at least one of *B. tribocorum, B. henselae, B. quintana* and *B. bacilliformis*.

b The number of *B. grahamii* genes with an ortholog in each of the other sequenced *Bartonella* genomes.

The *Bartonella* core genome, as defined by the number of predicted orthologs in all five species, consists of 904 genes. In addition to this conserved set of genes, we identified sixteen segments in a total of 732 kb in *B. grahamii* that are highly variable in comparison to the previously published *Bartonella* genomes, here called genomic islands (Table 2). Eight of these islands (BgGI 9–16) are located in close proximity to each other in a segment of 461 kb that contains 282 genes. The high density of islands indicates that this region is particularly susceptible to foreign DNA and we will refer to this highly atypical segment of the genome as the *chromosomal high plasticity zone*.

**Table 2:** Characteristics of genomic islands and prophages in *B. grahamii*.

Name	First gene^a^	Last gene^a^	Size	Genes^b^	Content
BgGI 1	Bgr_01430	Bgr_01610	61 kb	10	*Bartonella* adhesins
BgGI 2	Bgr_03020	Bgr_03830	60 kb	73	*Prophage Ia*
BgGI 3	Bgr_03980	Bgr_04180	23 kb	14	Membrane proteins
BgGI 4	Bgr_07490	Bgr_09310	166 kb	163	*Prophage Ib*, *fha*-repeats
BgGI 5	Bgr_09700	Bgr_10280	53 kb	55	Phage related
BgGI 6	Bgr_12450	Bgr_12590	20 kb	7	Membrane proteins
BgGI 7	Bgr_12750	Bgr_12950	24 kb	13	*fha*-repeat
BgGI 8	Bgr_13490	Bgr_13590	11 kb	5	Unknown
BgGI 9	Bgr_14480	Bgr_14750	40 kb	20	Autotransporters, phage
BgGI 10	Bgr_15090	Bgr_15450	43 kb	25	*fha*-repeat
BgGI 11	Bgr_15690	Bgr_15890	34 kb	16	*fha*-repeat
BgGI 12	Bgr_16030	Bgr_16480	70 kb	36	Autotransporters, phage
BgGI 13	Bgr_16650	Bgr_16880	32 kb	16	*Phage cluster II*
BgGI 14	Bgr_16900	Bgr_17120	37 kb	16	*fha*-repeat
BgGI 15	Bgr_17370	Bgr_17750	41 kb	36	*Phage cluster III*
BgGI 16	Bgr_17950	Bgr_18150	18 kb	17	Vbh T4SS
Prophage Ia	Bgr_03240	Bgr_03830	44 kb	56	Prophage
Prophage Ib	Bgr_08620	Bgr_09310	47 kb	67	Prophage

a The locus_tag of the first and the last gene in each genomic island.

b The number of predicted protein-coding genes in each genomic island.

#### High fraction of imported genes in the rodent-associated species

**Figure 2:**
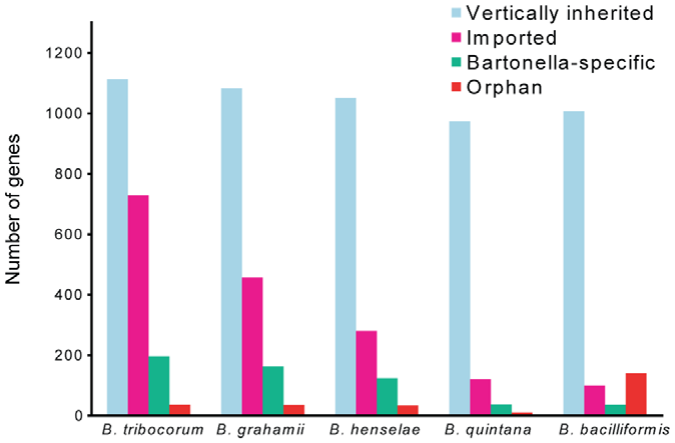
Gene classifications in the *Bartonella* genomes. Diagram showing the number of genes that are predicted as vertically inherited (from closely related alpha-proteobacterial species), imported (horizontally transferred to the ancestor of *Bartonella* or more recently), *Bartonella-*specific (only present in *Bartonella*), and orphans (only present in one species) in the sequenced *Bartonella* genomes.

A prediction of the total amount of horizontally transferred genes by phylogenetic analysis (excluding plasmid genes and hypothetical genes solely identified in *Bartonella*) classified 729 imported genes (35%) in *B. tribocorum* and 457 such genes (26%) in *B. grahamii* (Figure 2). The human-specific pathogens *B. quintana* and *B. bacilliformis* have the smallest genomes with less than 125 imported genes each (∼10%). Only a few genes were initially identified in *B. quintana* that were absent or pseudogenized in *B. henselae* [3]. Our analysis shows that these genes have homologs in the rodent-associated *Bartonella* species; substantiating the hypothesis that *B. quintana* has evolved by lineage-specific losses with no novel gene acquisitions [3]. *B. bacilliformis* contains a few imported genes solely identified in this lineage, including 15 copies of a hypothetical gene that is also present in *Leptospira interrogans.* However, because there are no known close relatives of *B. bacilliformis*, we cannot infer the direction of evolution (loss or gain) of genes specific to this species.

A functional dissection of the 457 imported genes in *B. grahamii* shows that one third code for bacteriophage proteins and most of the remaining for secretion systems, membrane proteins, transport proteins or hypothetical proteins (Table 3). The much higher number of imported genes in the rodent-associated lineage can be attributed both to novel genes and to a higher copy-number of genes that are also present in the other species. Specific to this lineage are for example genes for membrane proteins in the genomic island BgGI 3, which is located in a region where the other three genomes display undisrupted synteny (Figure S1). Also unique to the rodent-associated species are genes for ubiquinol-cytochrome *c* reductase and a hypothetical protein with similarity to a genomic island gene in *Photorhabdus luminescens,* which is present in seventeen and nine copies in *B. grahamii* and *B. tribocorum*, respectively. Many of the genes solely identified in *B. tribocorum* are located in a prophage present in four genomic islands.

**Table 3:** Function of imported genes in *B. grahamii.*

Function	Number of genes
Phage-related	165
T4SS	58
T5SS	35
Membrane proteins	30
Transport proteins	41
Hypothetical proteins	62
Other function	66
Total	457

### Expansion-Contraction of the Chromosomal High Plasticity Zone

#### Sequence similarity to auxiliary replicons

**Figure 3:**
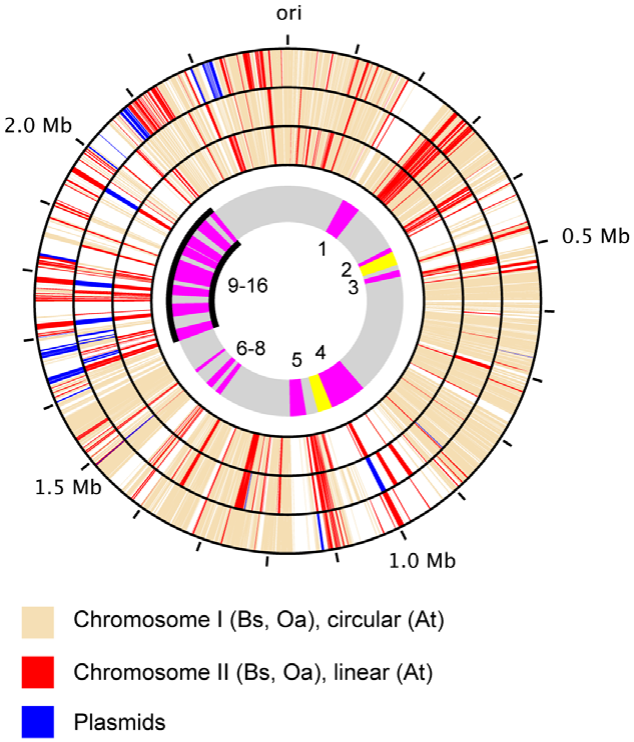
Sequence similarities to genes located on auxiliary replicons in other rhizobiales species. The innermost circle represents the genome of *B. grahamii*, with prophages in yellow, genomic islands in magenta and the chromosomal high plasticity zone in black. Numbers within the circle represent the genomic islands BgGI 1–16. The remaining circles show on which replicon the top Blast hit of each *B. grahamii* gene is located in each of *Brucella suis* (Bs; inner circle), *Ochrobactrum anthropi* (Oa; middle circle), and *Agrobacterium tumefaciens* (At; outer circle). The genome positions are shown on the outside, with 100 kb between each line.

Our analysis suggests dramatic changes in the mobile gene pool in the rodent- versus the human-associated *Bartonella* lineages. Because many of the expansion-contraction processes have occurred in the high plasticity zone, we first re-examined the hypothesis that this region was initially derived from the integration of an auxiliary replicon [3]. To this end, we blasted all *B. grahamii* genes against the genomes of *Brucella suis*, *Ochrobactrum anthropi* and *Agrobacterium tumefaciens,* three related species with one major chromosome and a large auxiliary replicon. We found that top hits (E<1e^−10^) to the auxiliary replicons were circa 2.5–3 times more frequent in the high plasticity zone, with most other such hits located within or in the vicinity of the genomic islands (Figure 3).

**Figure 4:**
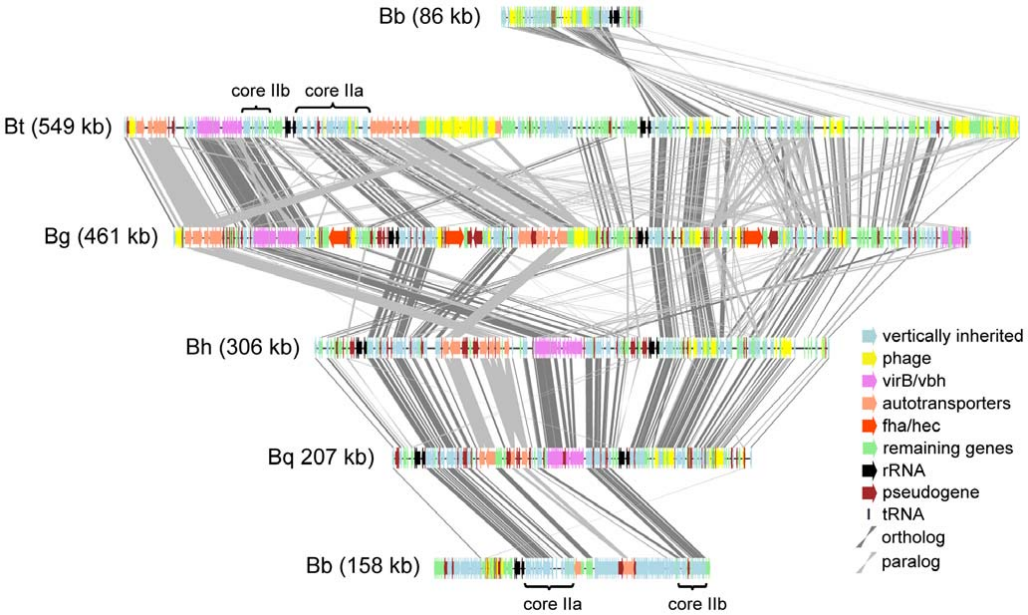
Comparison of the chromosomal high plasticity zone in the *Bartonella* genomes. Comparative gene map of the chromosomal high plasticity zone in the five sequenced *Bartonella* genomes. The locations of T4SS (*virB/vbh*) and T5SS (autotransporters, *fha/hec*) are shown. Species abbreviations are as in the legend to Figure 1. The total size of the region in each species is shown within parentheses. Due to the rearrangement around the replication origin in *B. bacilliformis,* the chromosomal high plasticity zone is divided into two parts, located on different sides of the origin.

A closer inspection of the high plasticity zone revealed two conserved segments, here called *core IIa* and *core IIb*, which are present in all sequenced *Bartonella* genomes (Figure 4). These segments are located between the two rRNA operons in *B. bacilliformis*, *B. henselae* and *B. quintana*, suggesting that this is the ancestral organization. In the rodent-associated species, the segment *core IIb* has been rearranged to a position immediately upstream of the first rRNA operon. Despite their conservation in *Bartonella*, the genes in *core IIb* have top hits to genes on auxiliary replicons, favoring their integration with an auxiliary replicon.

#### Acquisition and evolution of genes for secretion systems

Located between or near the conserved segments *core IIa* and *core IIb* are gene clusters for type IV secretion systems (T4SS) in all species except *B. bacilliformis,* and type V secretion systems (T5SS) in all species. In *B. grahamii*, we identified four operons for T4SS, one near *core IIb* (*virB*), one at the far end of the high plasticity zone (*vbh*-*1*), one downstream of the high plasticity zone (*trw*), and the fourth being plasmid-encoded (*vbh-2*). The T5SSs in the genomic islands of *B. grahamii* include fourteen genes for autotransporters, six copies of the two-component *fha/hec* operon and five copies of the *Bartonella* adhesin. The number of genes for T4SSs and T5SSs is highly variable across species, ranging from only eight genes in *B. bacilliformis* to more than one hundred in *B. grahamii* (Table 4).

**Table 4:** Number of genes involved in T4SS and T5SS in the sequenced *Bartonella* genomes.

Secretion system	Gene family	Bg	Bt	Bh	Bq	Bb
T4SS	*virB*	18	18	18	15	0
	*vbh*	22	10	0	0	0
	*trw*	29	30	23	23	0
	Total	69	58	41	38	0
T5SS	autotransporters	17	17	10	6	5
	*badA*	5	1	2	3	3
	*fha/hec*	13	8	7	0	0
	Total	35	26	19	9	8
All		104	84	60	47	8

Only full-length genes are included.

**Figure 5:**
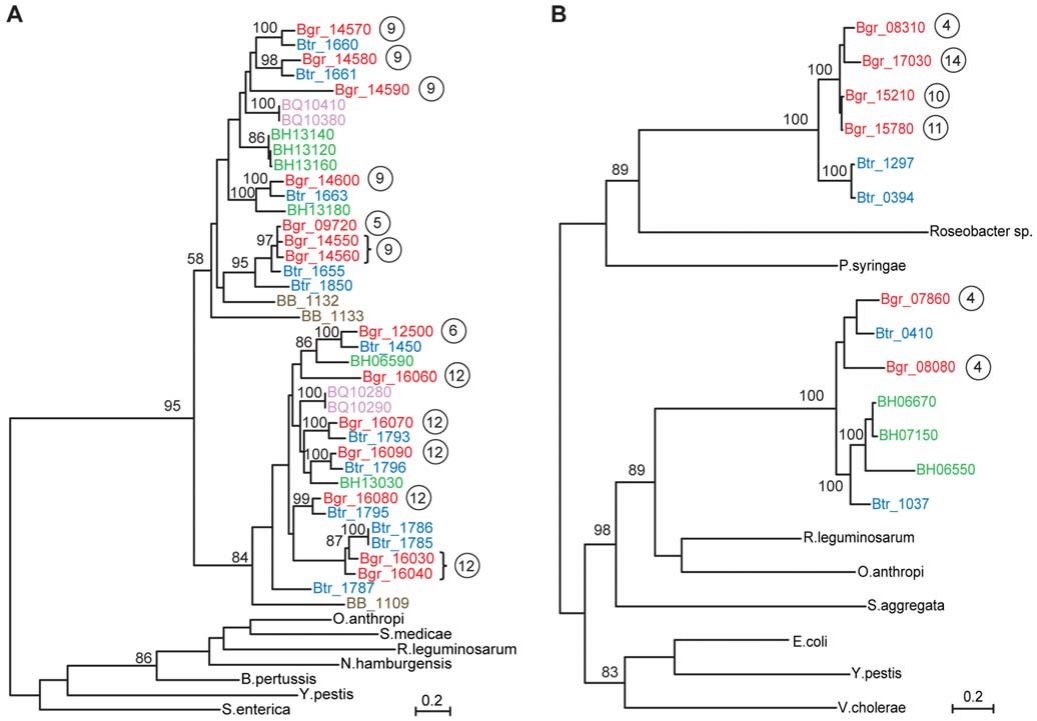
Phylogenetic analysis of genes for type V secretion systems. Phylogenetic trees of (A) autotransporters and (B) filamentous hemagglutinin. *Bartonella* genes are named with locus_tag (BARBAKC583 abbreviated as BB) and color-coded according to species: *B. grahamii* in red, *B. tribocorum* in blue, *B. henselae* in green, *B. quintana* in purple and *B. bacilliformis* in brown. After each *B. grahamii* gene, the genomic island in which the gene is located is indicated with numbers in circles. Branch lengths are according to maximum likelihood analysis and numbers represent bootstrap support values.

The autotransporters are ubiquitously present in all five *Bartonella* genomes and their genomic locations suggest that they were ancestrally positioned in between *core IIa* and *core IIb*. A phylogeny of the transmembrane domain of the autotransporters revealed two distinct *Bartonella* clades, both of which contain all species (Figure 5A). *B. bacilliformis* represents the earliest diverging lineage in both clades, suggesting that the acquisition event(s) extends back to the root of the *Bartonella* tree. Due to a rearrangement in the rodent-associated lineage, the autotransporter genes in the high plasticity zone of these species are physically separated into two clusters with 5–7 genes in each cluster. In *B. grahamii*, these genes are located in BgGI 9 and BgGI 12 (Figure 4). Their physical separation corresponds to their phylogenetic divergence, suggesting that each of the two clusters have expanded and contracted independently through a series of tandem gene duplication and/or deletion events.

The *virB* operon is conservatively placed between one cluster of autotransporter genes and *core IIb,* however, in the rodent-associated species this whole region has been translocated to a position immediately upstream of the first rRNA operon (Figure 4). The *virB* operon is absent from the *B. bacilliformis* genome and has been suggested to be acquired in a recent branch of the *Bartonella* tree [4]. Although homologs to the *virB* genes are circulating in the Rhizobiales auxiliary gene pool, the gene phylogenies do not match the species phylogeny, supporting horizontal gene acquisition [33].

Of the genes for secretion systems, the *fha/hec* gene cluster, which codes for filamentous hemagglutinin (FHA) and a hemolysin activator protein, is most labile in its presence/absence pattern. In the *B. grahamii* genome *fha/hec* is present in three copies in BgGI 4 and in one copy in BgGI 10, BgGI 11 and BgGI 14. The operon is located within a segment that is 7-fold repeated in the genome, hereafter referred to as the *fha-*repeat. The repeat is flanked by integrase and phage genes and varies in length from 10 to 28 kb, with nucleotide sequence identities ranging from 80 to 100%. Phylogenetic analysis of FHA reveals two distinct *Bartonella* clades (Figure 5B), both of which have their closest relative in other species, suggesting that the *fha/hec* genes in *Bartonella* were acquired twice independently. However, unlike the autotransporter genes, the *fha/hec* genes do not seem to have been located between the rRNA genes in the common ancestor, but were inserted into the high plasticity zone in *B. grahamii* very recently. Since the *fha/hec* operon, whenever present, is located in the proximity of phage genes, it might integrate, rearrange and excise at high frequencies.

### Bacteriophage Genes and Particles

#### Prophage genes

**Figure 6:**
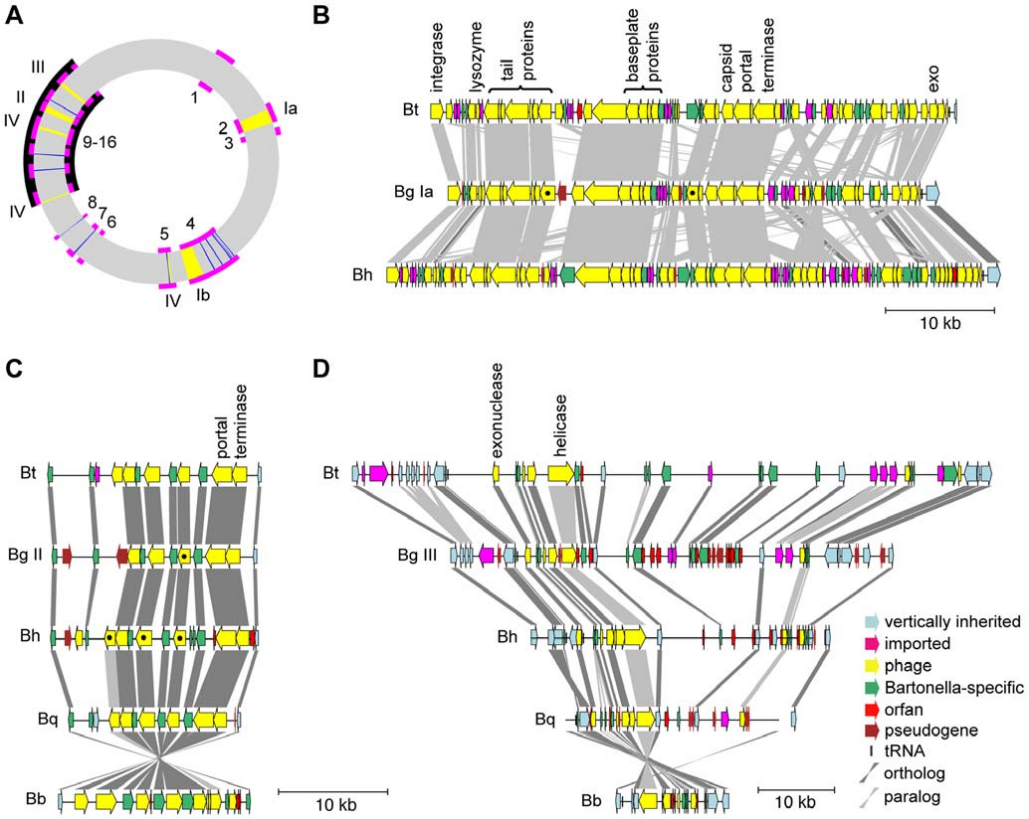
Phage clusters in *B. grahamii*. (A) Circular overview of the *B. grahamii* as4aup genome. The locations of phage clusters (yellow), genomic islands (magenta), the chromosomal high plasticity zone (black) and the gene with similarity to *S. thermophilus* phage Sfi18 (blue) are shown. Numbers outside the circle represent the phage clusters I-IV. Numbers within the circle represent the genomic islands BgGI 1–16. Detailed comparative gene maps are shown for (B) *prophage Ia,* (C) *phage cluster II*, and (D) *phage cluster III.* The color legend shown in (D) also applies to (B) and (C). Genes identified in mass spectrometry are marked with black dots. The putative origin of replication identified in the microarray analysis is located close to the helicase in (D).

Since phage related genes contribute much of the variability in *Bartonella*, we classified the most abundant such genes in *B. grahamii* into four phage clusters (Figure 6A), the largest of which is duplicated and referred to as *prophage Ia* (Figure 6B) and *prophage Ib*. This prophage has a size of circa 45 kb and contains one stretch of genes for capsid and portal proteins with similarity to phage lambda and another stretch encoding baseplate and tail proteins with similarity to phage P2. Many of the genes have homologs in prophages in *Vibrio harveyi*, *Wolbachia pipientis* and *Ochrobactrum anthrophi*, all with some conservation of gene order. The presence of genes in *B. bacilliformis* with similarity to those in *prophage I*, its absence in the closely related *Brucella* spp. and its equidistant relationship to unrelated species suggest that the prophage was acquired in an ancestor of the *Bartonella* species.

Several shorter clusters of phage genes may represent remnants of other integrated phages. Two such clusters, here called *phage cluster II* (Figure 6C) and *phage cluster III* (Figure 6D) are located in the chromosomal high plasticity zone and are well conserved in the *Bartonella* genomes. *Phage cluster II* includes genes for lambda-like phage portal and terminase proteins. Paralogs to some of these genes are also present in BgGI 4 and BgGI 5. *Phage cluster III* encodes a helicase with similarity to bacteriophage replication proteins and an exonuclease; these two conserved genes are located near to a region of mostly non-coding DNA that is variable in size across the *Bartonella* genomes.

*Phage cluster IV* contains genes for helicase (*dnaB*), phage late control protein D (*gpD*), phage tail X (*gpX*) and phage tail U (*gpU*) and is located adjacent to autotransporter genes in BgGI 5, BgGI 9 and BgGI 12. Among the sequenced *Bartonella* species, the genes in *phage cluster IV* are only present in *B. tribocorum,* where they belong to a prophage that is 35 kb in size and is similar to bacteriophage Mu. These results suggest that this phage integrated in the common ancestor of *B. grahamii* and *B. tribocorum*. Finally, *B. grahamii* has 29 copies of a gene with similarity to a gene from *S. termophilus* phage *Sfi18*, mostly in clusters of three genes within the *fha*-repeat.

#### Bacteriophage particles

**Figure 7:**
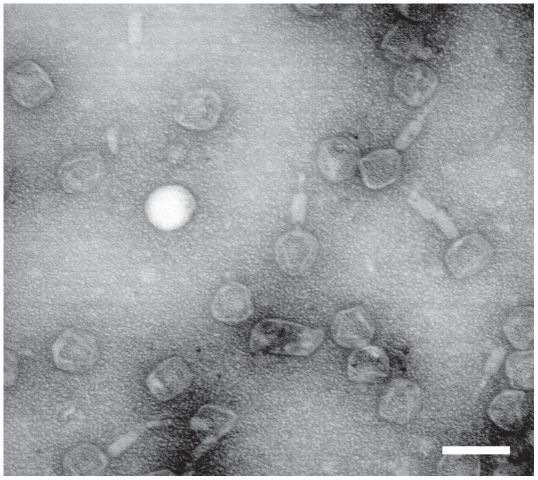
Bacteriophage particles from *B. grahamii*. Transmission electron microscopy of bacteriophage particles isolated from *B. grahamii* strain af165up. Round to icosahedral heads of 50–70 nm were observed both with and without tail, whereas there were few loose tails. The white bar is 100 nm.

We observed bacteriophage particles consisting of a round to icosahedral head of 50–70 nm in diameter and a sheathed tail of approximately 100 nm by transmission electron microscopy of bacteriophage preparations from *B. grahamii* strains af165up (Figure 7) and as4aup (Figure S2A). Many heads lacked tails, while there were few loose tails, suggesting the production of two morphologically different bacteriophage particles. Bacteriophage particles were also observed in *B. henselae* strain GreekCat-23 (Figure S2B), which was included for comparison, since it has been shown to lack *prophage I* [17].

We examined the protein profiles of the phage particles from all three strains using SDS-PAGE and mass spectrometry (Table S1). Two proteins from *prophage I*, corresponding to GpF1 and Gp20 (Figure 6B, genes with black dots), were identified in the *B. grahamii* strains, suggesting that *prophage I* is active in both these strains. The dominating protein in all strains was a phage related protein (Bgr_16730/BH14010) from *phage cluster II* (Figure 6C, genes with black dots). In *B. henselae* strain GreekCat-23 we also identified two other proteins from *phage cluster II*, corresponding to BH13960, a putative phage tail protein, and hypothetical protein BH13990 (Figure 6C, genes with black dots). The protein encoded by BH13960 has previously been observed in bacteriophage preparations from *B. henselae* and referred to as PapA [34].

Additionally, hemin binding protein A (HbpA), also known as Pap31, was present in bacteriophage preparations from *B. henselae* and *B. grahamii* grown on hematin agar plates. However, when *B. henselae* was cultivated in liquid medium in the absence of hemin, we were unable to identify HbpA among the extracted phage proteins (Table S1). This suggests that HbpA is not part of the bacteriophage, as has been suggested previously [35], but rather a bacterial outer membrane protein that is strongly expressed in the presence of hemin and co-precipitates with the phage particles.

**Figure 8:**
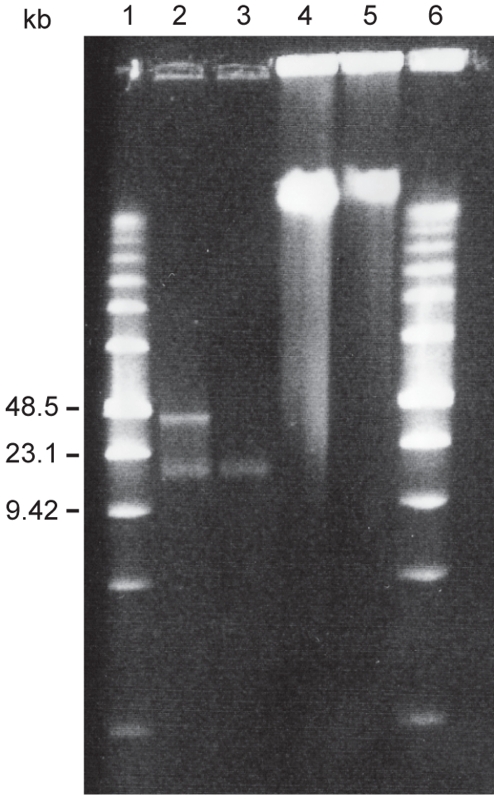
Bacteriophage DNA from *B. grahamii*. Agarose gel electrophoresis of *B. grahamii* bacteriophage DNA. Lane 1, Marker (Low Range PFG Marker, New England Biolabs); lane 2, phage DNA from *B. grahamii* af165up; lane 3, phage DNA from *B. grahamii* as4aup; lane 4, cellular DNA from *B. grahamii* af165up; lane 5, cellular DNA from *B. grahamii* as4aup; lane 6, marker. The sizes of the relevant bands of the marker are shown to the left.

We extracted bacteriophage DNA from all three strains, and agarose gel electrophoresis revealed two bands of circa 14 and 45 kb in *B. grahamii* strain af165up (Figure 8) and one band of circa 14 kb in *B. grahamii* strain as4aup (Figure 8) and *B. henselae* strain GreekCat-23 (Figure S3). While a 14 kb band has been observed in previous studies, this is to our knowledge the first report of a larger band in *Bartonella* phage DNA.

### Massive Amplification of the Chromosomal High Plasticity Zone

#### Amplification and packaging of the chromosomal high plasticity zone and *prophage I* into phage particles

**Figure 9:**
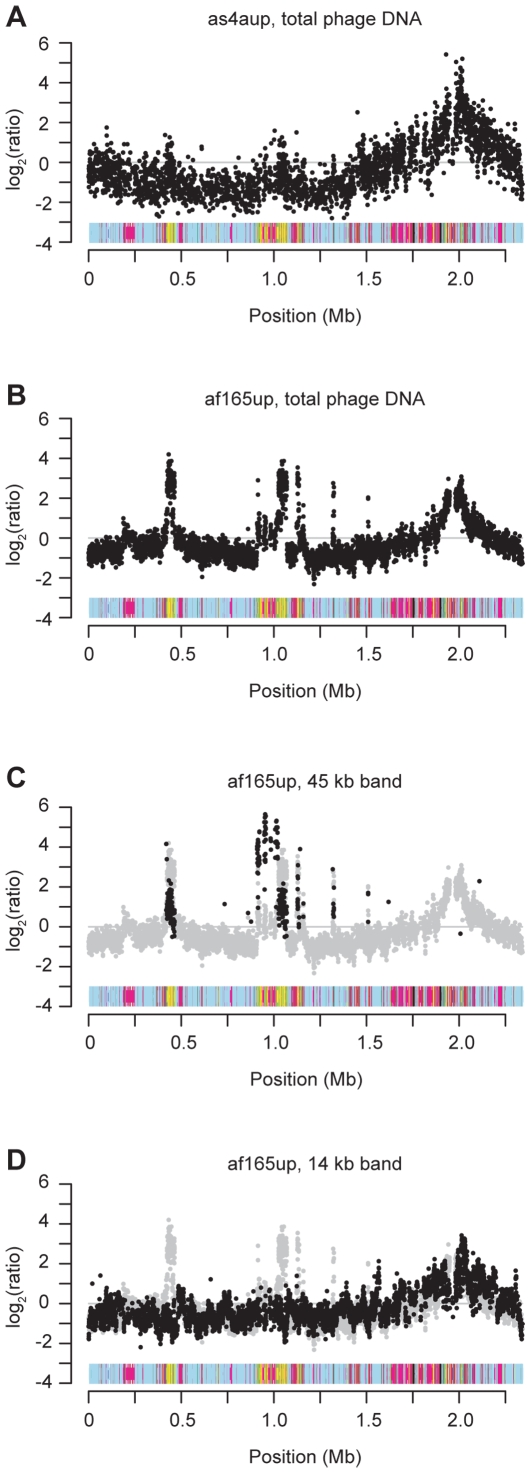
DNA content of *B. grahamii* bacteriophage particles. Results from microarray hybridizations of phage DNA versus cellular DNA of the same strain. The x-axis represents the genome of *B. grahamii* as4aup and the y-axis the hybridization signal (log_2_-ratio of phage DNA and cellular DNA). To exclude possible misinterpretation of repeated probes, probes with more than one exact match in the genome were not plotted unless located in the prophage regions. (A) Total phage DNA from *B. grahamii* as4aup. (B) Total phage DNA from *B. grahamii* af165up. Phage DNA extracted from (C) the 45 kb band and (D) the 14 kb band in agarose electrophoreses from *B. grahamii* af165up. Above the x-axis in all graphs is a representation of the genome of *B. grahamii* as4aup with the same color-coding as in Figure 1. In particular, phage genes are yellow. Grey dots in (C) and (D) show the result of the hybridization of total phage DNA from *B. grahamii* af165up (Figure 9B).

To examine the gene content of the bacteriophage DNA, we constructed a microarray from 4,438 oligomers of 60 bp in size, which covers 1703 protein-coding genes (96% of the predicted protein-coding genes in *B. grahamii*), 110 pseudogenes and 663 intergenic regions. We hybridized bacteriophage DNA from *B. grahamii* strains as4aup and af165up against cellular DNA from the same strain harvested at the same time point. In both strains, we observed an increase in hybridization signal over the chromosomal high plasticity zone and until *ori*, with the strongest signal in the centre and gradually decreasing in both directions (Figure 9AB). The peak, at which the signal is approximately 30-fold stronger in the phage DNA compared to the cellular DNA, is located close to or within the helicase in *phage cluster III* (Figure 6D). This site is homologous to the site where the amplification peak was observed in hybridizations of genomic DNA from *B. henselae* [17]. In strain af165up, we observed a strong amplification of *prophage I*, which is also visible in strain as4aup but to a much smaller extent.

To specifically assess the content of the 14 kb and the 45 kb bands in strain af165up, we extracted DNA from each band, and hybridized against cellular DNA. The results showed that the hybridization signal from the 45 kb band was concentrated to *prophage I* (Figure 9C), in agreement with the predicted size of this prophage. Although no 45 kb band was visible in phage DNA extracted from strain as4aup, we believe that *prophage I* is active also in this strain, albeit to a much lower extent, since proteins encoded by genes in this bacteriophage were identified in mass spectrometry, and this region was slightly over-represented in the total phage DNA. The 14 kb band contained DNA from the entire genome, with an over-representation of the amplified DNA from the chromosomal high plasticity zone (Figure 9D). This result is in agreement with previous descriptions of random or heterogenic DNA, and explains why others have experienced so many difficulties when trying to characterize this DNA.

#### The prophage and the chromosomal high plasticity zone are amplified and packaged into phage particles during all growth phases

**Figure 10:**
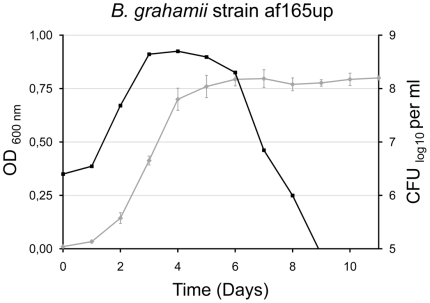
Growth curve of *B. grahamii* af165up. Growth curve of *B. grahamii* strain af165up in supplemented Schneider's medium. Bacterial growth was determined by measuring the OD_600_ in triplicates (grey line) and by quantifying the number of viable bacteria, expressed as CFU/ml (black line), at 24-h intervals. Samples were collected at the mid-logarithmic growth phase (day 2), the stationary phase (day 5), and at the end of the death phase (day 11).

**Figure 11:**
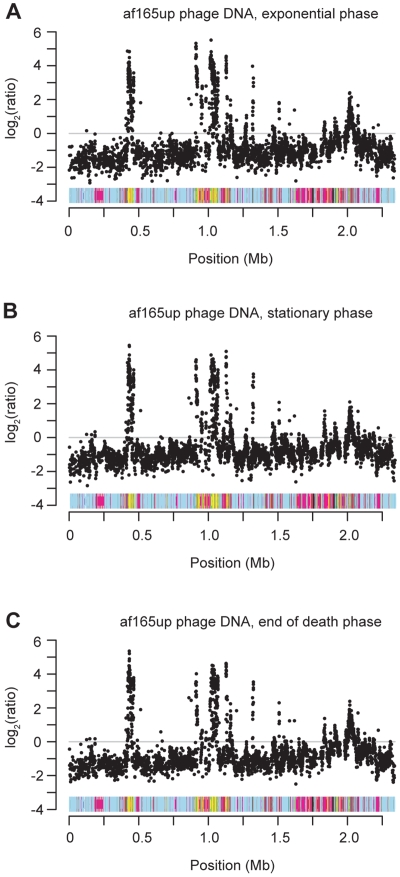
DNA content of *B. grahamii* bacteriophage particles isolated at different growth phases. Results from microarray hybridizations of DNA extracted from phage particles in (A) exponential phase (B) stationary phase and (C) end of death phase. To exclude possible misinterpretation of repeated probes, probes with more than one exact match in the genome were not plotted unless located in the prophage regions.

To examine whether induction of the prophage and run-off replication occur predominantly in dying cells, or possibly is the cause of bacterial death, we collected phage particles from three different time points from a liquid culture of *B. grahamii* strain af165up, selected to represent exponential growth phase (growing, viable), stationary phase (non-growing, viable) and death phase (non-growing, non-viable) (Figure 10). We extracted phage DNA from each time point to hybridize against chromosomal DNA extracted from the exponential growth phase. To obtain enough DNA for the hybridization experiments, we amplified the phage DNA using whole genome amplification. This procedure will reveal differences in the relative abundance of different genes in the phage versus the chromosomal DNA samples, but does not show differences in the total amount of phage DNA produced at the three growth stages. The results showed clearly that replication of *prophage I* is initiated already during the exponential growth phase with prophage DNA being over-represented in the phage particles at all three time points (Figure 11). Likewise, the products of run-off replication were present in relatively higher quantities in the isolated phage particles than in the preparations of chromosomal DNA at all time points.

## Discussion

In this study, we have resolved the long-standing debate about the content of the *Bartonella* bacteriophage particles. Altogether, our data suggests the presence of two distinct bacteriophages in *B. grahamii,* one of which is encoded by *prophage I* and packages its own DNA. The other bacteriophage, which is encoded by genes in *phage cluster II*, contains chromosomal DNA with an over-representation of DNA from the high plasticity zone. Bacteriophage particles that package random bacterial DNA rather than their own, with no lysis or other obvious negative consequences for the host cell are called gene transfer agents (GTAs). They are usually encoded by a segment of circa 15 kb that contains phage head and tail genes and they transfer DNA in 4–14 kb pieces [36]–[40]. These characteristics agree well with our observations, suggesting that the bacteriophage packaging the 14 kb band is a GTA. To our knowledge, this is the first demonstration of a GTA that packages chromosomal DNA that has first been partially amplified by run-off replication.

The identification of a GTA encoded by *phage cluster II* explains the previously puzzling observation of bacteriophage particles containing 14 kb DNA in many *Bartonella* species, including *B. quintana* and *B. bacilliformis,* that lack *prophage I*. A gene transfer agent, called RcGTA, which packages the genome in 4.5 kb pieces has previously been identified in the alpha-proteobacterial species *Rhodobacter capsulatus* [36]. Homologous gene clusters are widespread in the alpha-proteobacteria and the congruence in phylogenetic relationships inferred from rRNA and RcGTA genes suggests vertical transmission from a single GTA-containing ancestor [41]. However, no such genes could be identified in any of the *Bartonella* genomes, although most other members of the Rhizobiales contain partial and/or rearranged RcGTA-like gene clusters. The genes in *phage cluster II* also show no similarity to other known GTAs, suggesting that the *Bartonella* GTA is of a novel kind.

Despite the absence of sequence similarity between these two GTAs in the alpha-proteobacteria, their overall organization is similar, including genes coding for terminase, portal protein, and putative capsid and tail proteins. We identified gene clusters homologous to the *Bartonella* GTA in three recently sequenced genomes from the Rhizobiales species *Azorhizobium caulinodans, Methylobacterium radiotolerans* and *Rhodopseudomonas palustris* (Table S2), all of which also contain the RcGTA-like gene cluster. The scattered occurrence of the *Bartonella* GTA gene clusters in the alpha-proteobacteria indicates horizontal transmission.

Could run-off replication be initiated from a plasmid replication initiation site inherited from a once self-replicating megaplasmid? Such a phenomenon is believed to have occurred in the archaea, where multiple replication initiation sites have been demonstrated [42],[43], and suggested to have arisen from the capture of an extra-chromosomal plasmid-derived element [44]. Arguing against a plasmid-derived origin in *Bartonella* is that in the region close to the peak of the amplification we observed no sequence similarity to the *repABC* genes, which drives the replication and contains the *ori* in the megaplasmids of the Rhizobiales [45]. These genes are also not present anywhere else in the amplified region, providing no evidence for a megaplasmid-derived origin of replication.

It is more likely that replication starts from an origin derived from an inactivated prophage, as does escape replication in *Salmonella.* In this species, run-off replication can be induced in the laboratory by inactivating the integrase and/or excisionase genes [18], showing the ease with which a prophage can lose control of its own replication machinery. Capture of bacteriophage replication machineries has also been observed in mitochondria, where the nuclear genes for DNA polymerase and DNA primase-helicase (Twinkle) are derived from T7 bacteriophages [46]. It is interesting to note that the helicase that is located at the peak of run-off replication in *Bartonella* is homologous to Twinkle, supporting the hypothesis that the extra origin of replication is phage-derived. Homologous genes are present also in several other bacterial genomes, raising the possibility that capture of bacteriophage systems for bacterial replication may occur more frequently than recognized until now. However, no genes for the T7-like DNA or RNA polymerases could be found in the *Bartonella* genomes, suggesting that replication and transcription of the chromosomal high plasticity zone is not simply driven by a T7-like bacteriophage.

An important question is whether run-off replication and GTA-production are two independent phenomena, resulting from an error in the phage replication machinery and a degrading prophage, or whether the systems driving these processes co-evolve under selection and control of the bacterial genome. Several lines of evidence argue in favor of selection, particularly the conservation of *phage cluster III,* containing the origin of run-off replication, and *phage cluster II,* encoding the GTA, in all sequenced *Bartonella* genomes. Normally, prophages degrade rapidly as observed for *prophage I*, which has been lost independently from *B. quintana* and individual *B. henselae* strains [3],[17]. This is in analogy to the maintenance and vertical inheritance of the RcGTA-like gene cluster among several alpha-proteobacterial species, which has been attributed to selection [41].

What could be the selective advantage of linking run-off replication with a gene transfer agent? One advantage that we can think of is gene diversification by recombination and rapid spread of new gene variants. Because many genes for secretion systems are located in the amplified region, host adaptation and host switches could select for linkage of the two systems. Indeed, rapid diversification and horizontal transfer of host-adaptability genes within *Bartonella* populations have been demonstrated for the *trw* gene cluster [9]. Rodents in particular represent a large and genetically highly divergent host population, and multiple variants of surface proteins could help escape the immune system and promote interactions with a diverse set of host cell molecules. Under this hypothesis, it may be no coincidence that the rodent-associated *Bartonella* genomes contain the highest number of genes for secretion systems.

If run-off replication and production of GTAs are driven by selection, what are the cellular mechanisms and conditions responsible for regulating these processes? Our observation that the *Bartonella* GTAs containing amplified DNA are present already during exponential growth phase suggests that these genes are not induced to escape stressed or dying cells, nor that the GTAs are the cause of lysis and cell death. The relatively higher abundance of the products of run-off replication observed previously at later growth phase in *B. henselae* [17] is probably due to a gradual accumulation of amplified DNA with time. We can also exclude the possibility that run-off replication is regulated by *prophage I*, in trans, since *B. henselae* strain GreekCat-23, which lacks this prophage, still induces run-off replication [17]. Although the *Bartonella* GTA may very well be under the control of bacterial genes, the regulatory circuits involved are probably different from the growth-dependent production of RcGTA by histidyl-aspartyl signaling proteins [36] as well as by quorum sensing through long-chain acyl-homoserine lactones [47].

An exciting avenue for future work is to investigate how expression of the *Bartonella* GTA is regulated and whether run-off replication is regulated by the same control systems. It would also be interesting to determine where and when run-off replication and bacteriophage production occur within the rodent host and its fleas and whether different genome variants are generated during the infectious process. The availability of both the *B. grahamii* and the mouse genome also provides a technical platform that will enable future studies on the co-evolution of bacterial and host genes in natural mouse populations.

## Materials and Methods

### Bacterial Strains and Culture Conditions

*Bartonella grahamii* strains as4aup and af165up were isolated from a wood mouse and a yellow-necked mouse, respectively, captured in the vicinity of Uppsala in central Sweden [25]. *Bartonella henselae* strain GreekCat-23 was collected from a cat in the Thessaloniki area in Greece by Aphrodite Tea. The *Bartonella* strains were routinely grown on hematin agar plates in a humidified 5% CO_2_ incubator at 35°C. For growth in liquid culture, plate-grown bacteria were inoculated into Schneider's insect medium (Sigma) supplemented with 10% FBS and 5% sucrose as described by Riess *et al.* [48], additionally supplemented with 25 mM HEPES to keep the pH near 7. Numbers of viable bacteria were determined as colony-forming units (CFU) by plating 10-fold serial dilutions.

### Whole Genome Sequencing

Starting from single colonies, *B. grahamii* strain as4aup was cultured on hematin agar plates for five days. Genomic DNA was randomly sheared by nebulization and 1–3 kb sized fragments were recovered. The extracted fragments were cloned into a modified M13 vector using the ‘double adaptor’ method [49]. After 7–8 hours propagation in *E. coli,* ssM13 DNA was prepared for direct sequencing using Multi Screen MABCN1250 filter plates from Millipore combined with sodium-perchlorate lysis. Direct sequencing of the inserts was performed using DYEnamic ET Terminator Cycle Sequencing Kit (Amersham) on the MegaBACE 1000 DNA Analysis System according to the manufacturer's instructions. Cycle sequencing reaction cleanup was performed with the AutoSeq 96 Dye Terminator Clean-up Kit from Amersham.

A total of 31,166 shotgun sequences were obtained from the M13 library of *B. grahamii*. After assembly using the Phred and Phrap software [50],[51], physical gaps were closed by short and long-range PCR using primers from contig ends. Long-range PCR products were sheared by nebulization and end repaired with DNA Terminator End Repair Kit from Lucigen and blunt-end cloned into pcSmartHCKan vectors using the Clonesmart cloning kit and *E. coli* XL2-Blue Ultracompetent Cells from Stratagene. Recombinant colonies were grown in 50 µl 2xYT/kanamycin at 37°C overnight without shaking. 1 µl of the culture was used for amplification with TempliPhi Amplification Kit (GE-Healthcare). Both ends of the inserts were sequenced as described above. All ambiguous sites were manually edited with Consed [52] by re-sequencing of PCR products when necessary. A number of polymorphic sites in the *badA* region remain unresolved, these may well represent authentic polymorphisms since this gene is known to evolve very rapidly but we cannot exclude the possibility that there should be an additional *badA* gene.

### Southern Blotting and Assembly Verification

Bacteria were grown on hematin agar plates for 10 days. Prior to DNA extraction, the cells were suspended in TNE buffer (10 mM TRIS pH 8.0, 150 mM NaCl and 1 mM EDTA) and centrifuged; washes were repeated twice. DNA was isolated in agarose plugs, made using 2% SeaPlaque GTG agarose (Cambrex Bio Science, ME, USA) in 0.5× TBE buffer as described in [53], with minor modifications. One mm thick slices of DNA-containing plugs were digested with 10 U of *NotI*, *AscI*, *RsrII*, *SbfI* (New England Biolabs) and *SgfI* (Promega) restriction endonucleases separately, after 30 min pre-equilibration in TBE buffer on ice and subsequent 30 min pre-equilibration with an appropriate restriction buffer. Restriction was performed overnight at recommended conditions. The DNA fragments were separated in 0.9% PFGE-grade agarose (SeaKem Gold; Cambrex Bio Science) in 0.5× TBE buffer in the GenNavigator System apparatus (Amersham Biosciences) at 14°C and 5.6 V/cm, for a total of 65 hours. The total run was separated in six phases, using switch times ramped from 5 to 150 s. The sizes of the fragments were estimated using PFGE l-ladder and Yeast Chromosome PFG marker (New England Biolabs). Thirty-seven DNA fragments serving as probes were amplified, purified and labeled as described earlier [17]. Southern blotting, hybridization and signal detection were performed as described earlier [17].

*NotI* restriction digest was used to verify the assembly of the B. grahamii genome. DNA was digested and separated as described above, using 1.1% low melting point SeaPlaque agarose for casting the gel. The gel was run and bands were excised as suggested by the protocol extraction of high molecular weight DNA from gel [54]. The blocks of gel containing individual bands of interest were excised and the agarose was digested using ß-agarase I (New England Biolabs) according to the manufacturer's instructions. From the reaction mix, DNA was extracted with equal volume of phenol: chloroform: isoamyl alcohol (25∶24∶1). The aqueous phase was cleaned up with equal volume of chloroform: isoamyl alcohol (24∶1), and DNA was precipitated with 2 volumes ice-cold isopropanol at −20°C overnight, washed with 70% ethanol twice and re-dissolved in TE buffer pH 7.5. 1 µg DNA from each of the two smallest *NotI* bands was hybridized against total genomic DNA to a microarray (see below), to see which part of the genome the band represented.

### Gene Prediction and Annotation

Curation and annotation of the genome were done with the annotation platform GenDB [55]. Protein-coding genes were predicted with GLIMMER [56] and CRITICA [57]. Similarity searches were performed against several databases, including GenBank (nt and nr), UNIPROT [58], KEGG [59] and COG [60]. Protein domains were identified with InterProScan [61] and the InterPro database [62]. SignalP [63], helix-turn-helix [64], and TMHMM [65] were used. RNA genes were identified with tRNA-Scan-SE [66] and ARAGORN [67].

### Gene Classification, Definition of Genomic Islands, and Phylogenetic Analysis

Orthologs between *Bartonella* species were predicted as reciprocal best Blastp [68] hits, excluding genes with top hits in the same species. Paralogs were predicted using blastclust (http://www.ncbi.nlm.nih.gov/Web/Newsltr/Spring04/blastlab.html), requiring 50% similarity over 40% of the length. Phage genes were defined based on Blast hits and literature.

Genes were defined as orphans if there was no convincing Blast hit in the GenBank nr database. Genes were defined as *Bartonella-*specific if the only hits were to other *Bartonella* species. For remaining genes, all orthologs and paralogs along with Blast hits from a selection of 27 completely sequenced alpha-proteobacterial genomes were used for phylogenetic analysis. To get a broad representation of species we further selected top Blastp hits up to a total of 40 genes (E<1e^−10^), excluding all other alpha-proteobacterial hits. Protein sequences were aligned with clustalW [69], and phylogenetic trees were inferred with PHYML [70], using the JTT model of amino acid substitution, fixed proportion of variable sites, one substitution rate category, estimated gamma distribution parameter and 100 bootstrap replicates. To get a rough estimation of the number of imported and vertically inherited genes, we inspected all trees with less than 18 Blast hits to the selected alpha-proteobacterial genomes manually. Imported genes were defined as those for which there was no indication of vertical inheritance from the ancestor of *Bartonella* and its close relatives. The genes for Type IV secretion systems were classified as imported despite being common in the alpha-proteobacteria, since previous analyses suggest a recent acquisition of these genes [33]. Gene classifications were transferred from *B. grahamii* to the other *Bartonella* species using best Blast hit (E<0.001). Genes in the other species without homologs in *B. grahamii* were manually classified.

Genomic islands were manually located in *B. grahamii* based on a synteny comparison with the previously published genomes (*B. tribocorum, B. henselae* and *B. quintana*). A genomic island was defined as a contiguous segment larger than 10 kb containing orphans, non-orthologous genes, or long non-coding regions. Island boundaries were extended to include adjacent *Bartonella*-specific genes or imported genes. For simplicity, boundaries of islands were always defined as the boundaries of the adjacent non-island genes.

Protein sequences for FHA and autotransporters were aligned with Kalign [71] and phylogenetic trees were inferred with RAxML [72], substitution model PROTMIXWAG, 100 bootstrap replicates. Since the autotransporter genes are of very variable length, only the conserved beta-domain, identified with HMMPfam in InterProScan [73], was used for phylogenetic inference.

### Isolation of Phage Particles, Extraction of Genomic and Phage DNA, and Mass Spectrometry

Bacterial cells were either harvested after 5 to 15 days of culture on hematin agar plates, or at selected time points during growth in liquid culture. While samples from liquid culture were collected directly, plate-grown bacteria were either overlaid with 5 ml Brain-Heart Infusion Broth (Difco) the day prior to collection or re-suspended in SM buffer. After centrifugation at ca. 4000×g for 15 min at 4°C, supernatants were collected, filtered through 0.2 µm filters and enriched using polyethylene glycol as described in [21]. Phage pellets were re-suspended in 30 µl TE buffer pH 8.0 or PBS.

Extraction of DNA from the bacterial pellets was performed using the AquaPure Genomic DNA Kit (Bio-Rad) according to the manufacturer's instructions. Phage DNA from plate cultures was extracted as described in [21]. Phage DNA from liquid culture was extracted in combination with amplification using the REPLI-g Mini kit (Qiagen) according to the manufacturer's protocol for DNA amplification from blood or cells. Concentrations were determined by measuring absorbance using a NanoDrop ND-1000.

Size selection of phage DNA fragments from non-amplified preparations was carried out by separation in 0.7% (w/v) low melting point agarose (SeaKem Plaque, Cambrex) gels containing ethidium bromide, in 1× TAE buffer at 4°C at 6 v/cm overnight. The fragments were visualized under near-UV light (λ = 360 nm) and excised with a sterile scalpel blade. Two volumes of ß-agarase I buffer (New England Biolabs) amended with 100 mM NaCl, 30 µM spermine and 70 µM spermidine [58] were added to each slice, followed by equilibration on ice three times for 30 min. Gel slices were melted at 65°C for 15 min, cooled to 42°C for 10 min and digested with 2 U of ß-agarase I (New England Biolabs) for one hour. ß-agarase was deactivated at 95°C for 5 minutes and the DNA was extracted by equal volume of phenol-chloroform-isoamyl alcohol (25∶24∶1) as described in [54]. DNA from the 14 kb band of *B. grahamii* af165up was amplified using the REPLI-g Mini kit.

The protein components of the bacteriophage particles were separated by SDS-PAGE (12%) after being heat-denaturized at 95°C for 5 minutes. Mass spectrometry was performed for each band. Proteins were identified based on computational searches in the NCBI non-redundant (nr) database and a local database consisting of the *B. grahamii* protein sequences.

### Microarray Design and Hybridization

We designed 4438 60-mer oligonucleotides, representing 1703 protein-coding genes, 110 pseudogenes and 663 spacers with OligoArray2.1 [74]. A probe was considered to belong to a gene if at least half the probe was located within the predicted borders of the gene. The probes were spotted in three replicates on Ultra-GAPS-coated slides (Cornings, Inc.). Slides were cross-linked at 250 mJ/cm^2^, and prehybridized at 42°C for 45–60 min in 3X SSC, 0.1% SDS and 0.1 mg/ml bovine serum albumin. Labeling of genomic DNA was performed as in [75]. Hybridizations were performed at 42°C overnight in hybridization solution containing 5X SSC, 0.1% SDS, 0.1 mg/ml sonicated salmon sperm DNA and 20% formamide.

### Analysis of Microarray Data

Scanning and image analysis were performed with a GenePix 4100A scanner and the GenePix 5.1 software (Axon Instruments, Molecular Devices) as described previously [17]. Phage and bacterial DNA are referred to as Ch1 and Ch2 respectively, and spots meeting the following criteria were removed from further analysis: Spots flagged as bad or not found during quantification, spots with more than 5% saturated pixels in either channel, Ch1 spot median below 3 times the Ch1 background median, less than 95% of pixels having a Ch1 intensity higher than background intensity plus one standard deviation, less than 90% of pixels having a Ch1 intensity higher than background intensity plus two standard deviations or spots having less than 70 pixels. M values were computed as log_2_(Ch1/Ch2).

### Accession Numbers

The genome sequence of *B. grahamii* has been deposited in GenBank under the accession numbers CP001562 for the chromosome and CP001563 for pBGR3. The microarray data have been deposited in the ArrayExpress database of the European Bioinformatics Institute under the accession numbers A-MEXP-1576 for the array design and E-MEXP-2149 for the experimental data.

## Supporting Information

Figure S1 Comparative gene map of the genomic region containing BgGI 1.
(0.27 MB PDF)

Figure S2 Electron micrographs of bacteriophage particles isolated from cultivations with *B. grahamii* and *B. henselae*. (A) Phage particles isolated from *B. grahamii* as4aup were observed both with and without tails. (B) Phage particles isolated from *B. henselae* GreekCat-23 without visible tails. The black bars are 100 nm.
(0.34 MB PDF)

Figure S3 PFGE migration of undigested DNA from bacteriophage particles isolated from *B. henselae* GreekCat-23. The arrow indicates a 14 kb DNA band. Lane 1, low-range PFGE marker; lane 2, empty; lane 3, lambda mix marker; lane4, GreekCat-23 bacteriophage DNA.
(0.13 MB PDF)

Table S1 Proteins identified in mass spectrometry of bacteriophage preparations from *B. grahamii* and *B. henselae*.
(0.06 MB PDF)

Table S2 Rhizobiales species with homologs to the genes in *phage cluster II*.
(0.07 MB PDF)
