# Supplementary figures and images for: Run-Off Replication of Host-Adaptability Genes Is Associated with Gene Transfer Agents in the Genome of Mouse-Infecting Bartonella grahamii
Source: PLoS Genet. 2009 Jul 3;5(7):e1000546. doi: 10.1371/journal.pgen.1000546 (PMC2697382; doi:10.1371/journal.pgen.1000546)

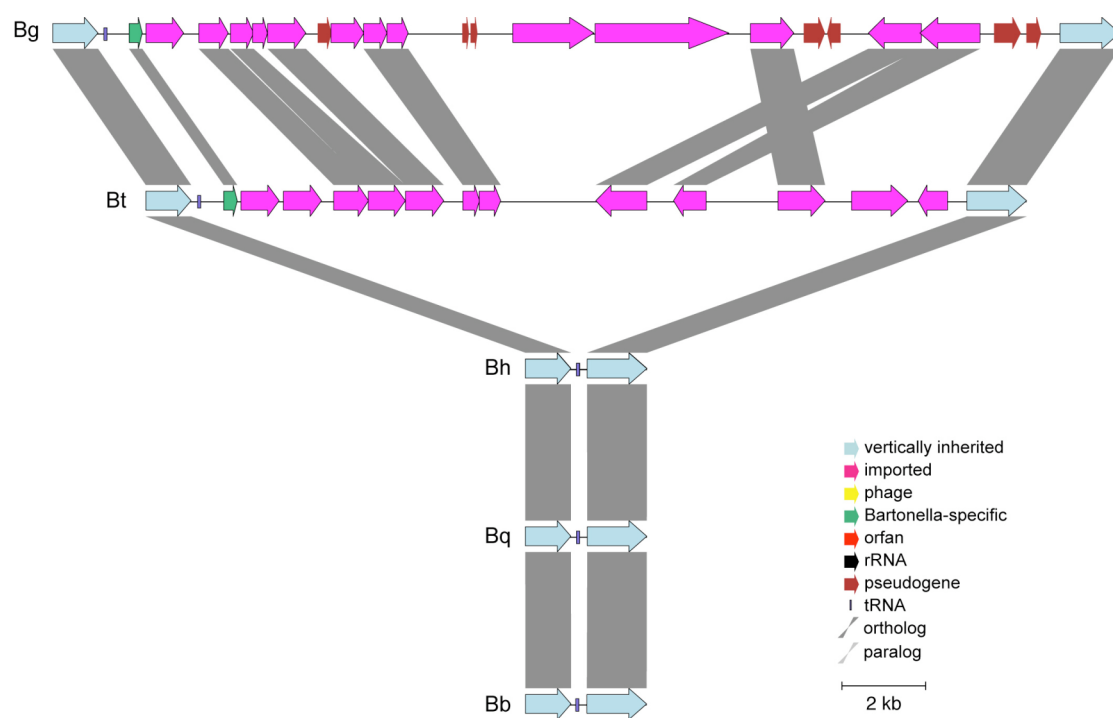

**Figure S1.** Comparative gene map of the genomic region containing BgGI 1.

Supplement: Figure S1 — Comparative gene map of the genomic region containing BgGI 1. (0.27 MB PDF) [file pgen.1000546.s001.pdf]
