# Supplementary material for: Run-Off Replication of Host-Adaptability Genes Is Associated with Gene Transfer Agents in the Genome of Mouse-Infecting Bartonella grahamii
Source: PLoS Genet. 2009 Jul 3;5(7):e1000546. doi: 10.1371/journal.pgen.1000546 (PMC2697382; doi:10.1371/journal.pgen.1000546)
Supplement: Figure S2 — Electron micrographs of bacteriophage particles isolated from cultivations with B. grahamii and B. henselae. (A) Phage particles isolated from B. grahamii as4aup were observed both with and without tails. (B) Phage particles isolated from B. henselae GreekCat-23 without visible tails. The black bars are 100 nm. (0.34 MB PDF) [file pgen.1000546.s002.pdf]

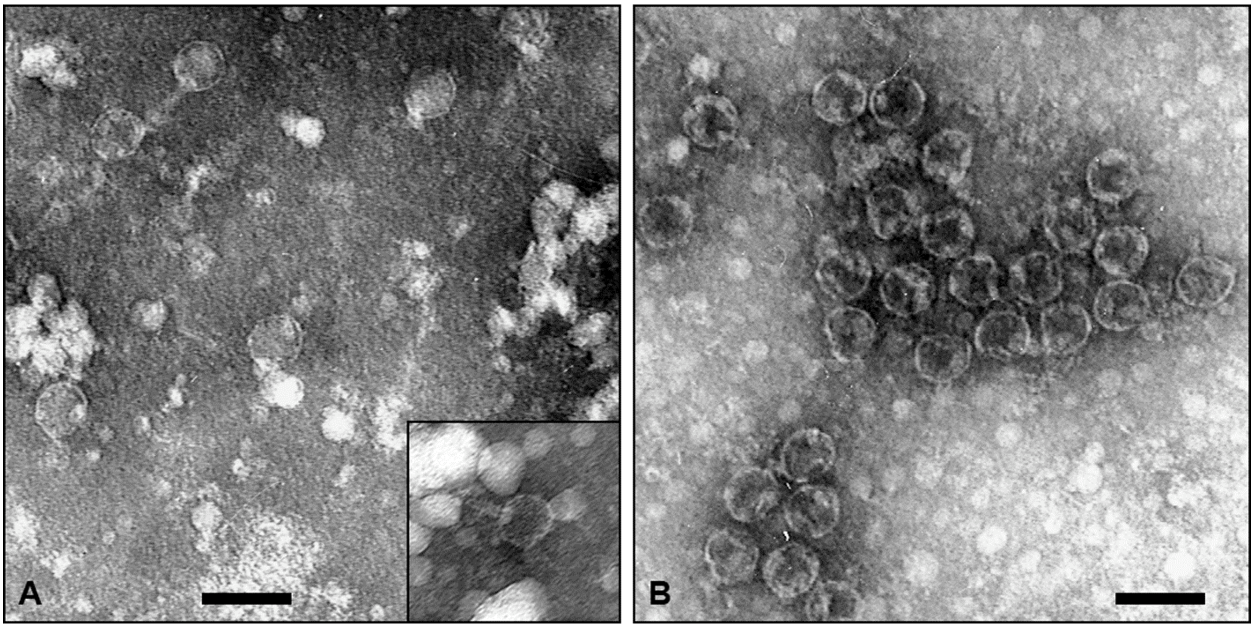

Figure S2. Electron micrographs of bacteriophage particles isolated from cultivations with *B. grahamii* and *B. henselae*. (A) Phage particles isolated from *B. grahamii* as4aup were observed both with and without tails. (B) Phage particles isolated from *B. henselae* GreekCat-23 without visible tails. The black bars are 100 nm.
