# Supplementary material for: Run-Off Replication of Host-Adaptability Genes Is Associated with Gene Transfer Agents in the Genome of Mouse-Infecting Bartonella grahamii
Source: PLoS Genet. 2009 Jul 3;5(7):e1000546. doi: 10.1371/journal.pgen.1000546 (PMC2697382; doi:10.1371/journal.pgen.1000546)
Supplement: Figure S3 — PFGE migration of undigested DNA from bacteriophage particles isolated from B. henselae GreekCat-23. The arrow indicates a 14 kb DNA band. Lane 1, low-range PFGE marker; lane 2, empty; lane 3, lambda mix marker; lane4, GreekCat-23 bacteriophage DNA. (0.13 MB PDF) [file pgen.1000546.s003.pdf]

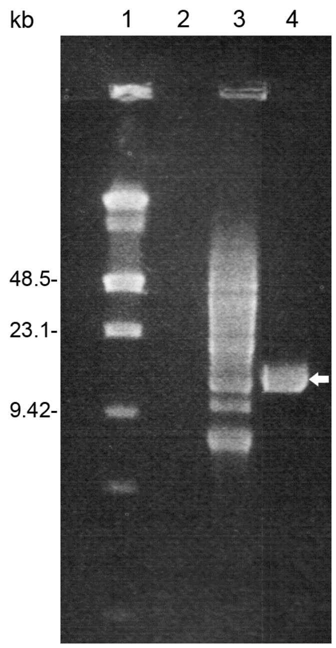

Figure S3. PFGE migration of undigested DNA from bacteriophage particles isolated from *B. henselae* GreekCat-23. The arrow indicates a 14 kb DNA band. Lane 1, low-range PFGE marker; lane 2, empty; lane 3, lambda mix marker; lane4, GreekCat-23 bacteriophage DNA.
