# Supplementary material for: Run-Off Replication of Host-Adaptability Genes Is Associated with Gene Transfer Agents in the Genome of Mouse-Infecting Bartonella grahamii
Source: PLoS Genet. 2009 Jul 3;5(7):e1000546. doi: 10.1371/journal.pgen.1000546 (PMC2697382; doi:10.1371/journal.pgen.1000546)
Supplement: Table S1 — Proteins identified in mass spectrometry of bacteriophage preparations from B. grahamii and B. henselae. (0.06 MB PDF) [file pgen.1000546.s004.pdf]

Table S1. Proteins identified in mass spectrometry of bacteriophage preparations from *B. grahamii* strains as4aup and af165up and *B. henselae* strain GreekCat-23. Sizes are based on estimations from SDS-PAGE, and should be regarded as rough. For *B. henselae* genes, the locus_tag of the most similar gene in *B. grahamii* is shown within parentheses.

B. grahamii strain as4aup grown on hematin agar plates.

Size (kDa)	Locus_tag	Annotation	Note
50	Bgr_03370/Bgr_08800	phage tail sheath protein FI	*prophage I*
47	Bgr_16730	phage related protein	*phage cluster II*
47	Bgr_16380	outer membrane protein	bacterial protein?
44	Bgr_03530/Bgr_09020	phage protein Gp20	*prophage I*
34	Bgr_07490	hemin binding protein A	bacterial protein?
32	Bgr_02670/Bgr_02650	hemin binding protein A	bacterial protein?

B. grahamii strain af165up grown on hematin agar plates.

Size (kDa)	Locus_tag	Annotation	Note
47	Bgr_03370/Bgr_08800	phage tail sheath protein FI	*prophage I*
47	Bgr_16730	phage related protein	*phage cluster II*
44	Bgr_03530/Bgr_09020	phage protein Gp20	*prophage I*
34	Bgr_07490	hemin binding protein A	bacterial protein?
32	Bgr_02670/Bgr_02650	hemin binding protein A	bacterial protein?

B. henselae strain GreekCat-23 grown on hematin agar plates.

Size (kDa)	Locus_tag	Annotation	Note
47	BH13990 (Bgr_16710)	phage related protein	*phage cluster II*
35	BH02560 (Bgr_02650)	hemin binding protein A	bacterial protein?

B. henselae strain GreekCat-23 grown in liquid medium in the absence of hemin.

Size (kDa)	Locus_tag	Annotation	Note
54	BH13990 (Bgr_16710)	hypothetical protein	*phage cluster II*
47	BH14010 (Bgr_16730)	phage related protein	*phage cluster II*
40	BH13960 (Bgr_16680)	putative phage tail protein	*phage cluster II*
