# Supplementary material for: Run-Off Replication of Host-Adaptability Genes Is Associated with Gene Transfer Agents in the Genome of Mouse-Infecting Bartonella grahamii
Source: PLoS Genet. 2009 Jul 3;5(7):e1000546. doi: 10.1371/journal.pgen.1000546 (PMC2697382; doi:10.1371/journal.pgen.1000546)
Supplement: Table S2 — Rhizobiales species with homologs to the genes in phage cluster II. (0.07 MB PDF) [file pgen.1000546.s005.pdf]

Table S2. Rhizobiales species with homologs to genes in *phage cluster II*. The locus_tag and the e-value (Blastp against nr) are shown.

Species	Bgr_16690	Bgr_16700	Bgr_16710	Bgr_16720	Bgr_16730	Bgr_16740	Bgr_16750	Bgr_16760
Azorhizobium caulinodans ORS 571	AZC_0849 6e-12	AZC_0848 0.71	AZC_0846 8e-67	AZC_0845 1e-08	AZC_0843 3e-95	AZC_0841 8e-11	AZC_0840 e-128	
Methylobacterium radiotolerans JCM	Mrad2831_5683 5e-06	Mrad2831_5684 6.0	Mrad2831_5686 1e-40	Mrad2831_5688 3e-07	Mrad2831_5690 2e-83	Mrad2831_5694 2e-04	Mrad2831_5695 e-118	Mrad2831_5696 1e-55
Rhodopseudomonas palustris TIE-1	RpalDRAFT_1706 0.003		RpalDRAFT_1704 8e-58	RpalDRAFT_1703 7e-10	RpalDRAFT_1701 4e-83	RpalDRAFT_1700 5e-05	RpalDRAFT_1697 e-125	RpalDRAFT_1695 e-145
